# *catena*-Poly[zinc-tris­(μ-dimethyl­carbamato-κ^2^ *O*:*O*′)-zinc-μ-(2-phenyl­benzimidazolido-κ^2^ *N*:*N*′]

**DOI:** 10.1107/S1600536811053177

**Published:** 2011-12-17

**Authors:** Mark A. Rodriguez, Dorina F. Sava, Tina M. Nenoff

**Affiliations:** a PO Box 5800, MS 1411, Sandia National Laboratories, Albuquerque, NM 87185, USA; b PO Box 5800, MS 1415, Sandia National Laboratories, Albuquerque, NM 87185, USA

## Abstract

The crystal structure of the title compound, [Zn_2_(C_13_H_9_N_2_)(C_3_H_6_NO_2_)_3_]_*n*_, displays a long chiral chain. This is composed of zinc-dimer clusters capped by dimethyl­carbamate ligands, which lie on crystallographic twofold rotation axes and are polymerically linked in one dimension by 2-phenyl­benzimidadole (2–PBImi) organic ligands. The two Zn^2+^ ions defining the dimetal cluster are crystallographically independent, but display very similar coordination modes and tetra­hedral geometry. As such, each Zn^2+^ ion is coordinated on one side by the N-donor imidazole linker, while the other three available coordination sites are fully occupied by the O atoms from the capping dimethyl­carbamates. The chirality of the chain extends along the *c* axis, generating a rather long 52.470 (11) Å cell axis. Inter­estingly, the chiral material crystallizes from completely achiral precursors. A twofold axis and 3_1_ screw axis serve to generate the long asymmetric unit.

## Related literature

For the structure of another zinc–adeninate compound, see: An *et al.* (2009 ▶). This structure, formed with adenine, contains a similar but not identical ligand as that of the 2-PBImi mol­ecule. Inter­estingly, this Zn-adeninate structure also displays the presence of dimethyl­carbamate, but in the case of the zinc-adeninate it is not a bridging mol­ecule between Zn^2+^ cations, but is terminally tethered to the Zn^2+^ ions. The dimethyl­carbamate capping mol­ecules formed *in situ* during the synthesis; there is precedence for such *in situ* reactions (An *et al.* 2009 ▶; Dell’Amico *et al.* 2003 ▶).
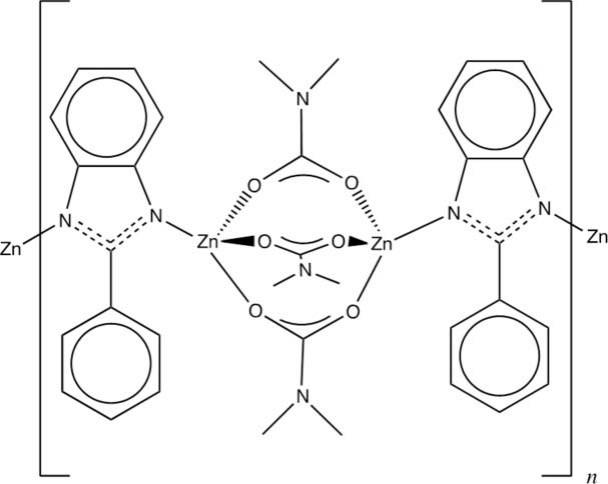


## Experimental

### 

#### Crystal data

[Zn_2_(C_13_H_9_N_2_)(C_3_H_6_NO_2_)_3_]
*M* *_r_* = 588.23
Trigonal, 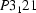

*a* = 9.0521 (13) Å
*c* = 52.470 (11) Å
*V* = 3723.4 (11) Å^3^
*Z* = 6
Mo *K*α radiation
μ = 1.98 mm^−1^
*T* = 188 K
0.20 × 0.19 × 0.15 mm

#### Data collection

Bruker APEX CCD diffractometer
Absorption correction: numerical (*SADABS*; Sheldrick, 1996 ▶) *T*_min_ = 0.681, *T*_max_ = 0.742
26966 measured reflections
4386 independent reflections
4132 reflections with *I* > 2σ(*I*)
*R*_int_ = 0.044

#### Refinement

*R*[*F* ^2^ > 2σ(*F* ^2^)] = 0.029
*wR*(*F* ^2^) = 0.059
*S* = 1.09
4386 reflections
333 parameters
H-atom parameters constrained
Δρ_max_ = 0.25 e Å^−3^
Δρ_min_ = −0.24 e Å^−3^
Absolute structure: Flack (1983 ▶), 1754 Friedel pairs
Flack parameter: 0.011 (12)

### 

Data collection: *SMART* (Bruker, 2007 ▶); cell refinement: *SAINT* (Bruker, 2007 ▶); data reduction: *SAINT*; program(s) used to solve structure: *SHELXTL* (Sheldrick, 2008 ▶); program(s) used to refine structure: *SHELXTL*; molecular graphics: *XSHELL* (Bruker, 2007 ▶) and *Mercury* (Macrae *et al.*, 2008 ▶); software used to prepare material for publication: *SHELXTL*.

## Supplementary Material

Crystal structure: contains datablock(s) I, global. DOI: 10.1107/S1600536811053177/nk2124sup1.cif

Structure factors: contains datablock(s) I. DOI: 10.1107/S1600536811053177/nk2124Isup2.hkl

Additional supplementary materials:  crystallographic information; 3D view; checkCIF report

## Figures and Tables

**Fig. 1.**
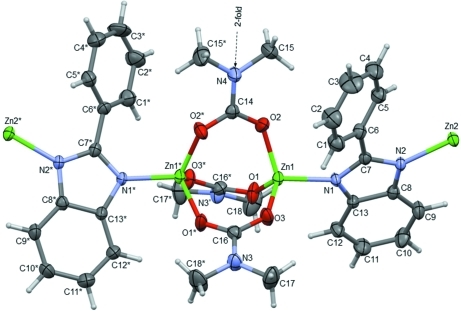
A view of the molecular building block (MBB) for the title compound, with labels and 50% probability displacement ellipsoids for non-H atoms. A twofold rotation axis extends through the polymeric chain parallel to the N4—C14 bond (and coincident with the a axis) and relates atoms by symmetry. For clarity of the MBB, dimethylcarbamate molecules attached to the Zn2 metal center have been removed. Atom labels containing asterisks indicate symmetry equivalent atoms.

**Fig. 2.**
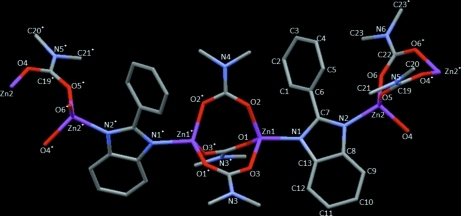
Capped stick image of the entire asymmetric unit for the title compound. This plot shows the second set of demethylcarbamate molecules which coordinate to the Zn2 atom. Atom labels containing asterisks indicate symmetry equivalent atoms.

**Fig. 3.**
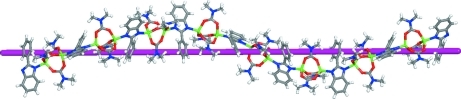
A ball-and-stick representation of a single chiral chain for the title compound. The chain propagates in the c axis direction.

## supplementary crystallographic information

### Comment

This manuscript documents possibly the first reported structure of the linker 2-phenylbenzimidazole (2–PBImi), as well as a coordination polymer derived from this molecule. The 2-PBImi molecule was investigated as a possible linker molecule for the synthesis of metal-organic framworks (MOFs). As a result of this research, the title compound was observed. This polymeric chain structure is derived from two tetrahedral metal centers that are capped by three dimethylcarbamates, resulting in a very rare (novel) molecular building block (MBB). There are a few published examples that display similar environments (see An, *et al.*, 2009), but none display identical coodination. The dimethylcarbamate capping molecules formed *in situ* during the synthesis; there is precedence for such *in situ* reactions (An, *et al.* 2009; Dell'Amico, *et al.* 2003).

Figure 1 shows the MBB for the chain. A cluster composed of two Zn cations bridged by three dimethylcarbamate molecules is bracketed on either side by 2-PBImi linkers to complete the tetrahedrally coordinated Zn1 cations. A twofold axis (coincident with the *a* axis direction) is also shown to illustrate how the atoms in the MBB are related by symmetry. Atoms with asterisks indicate symmetry equivalent atoms within the MBB. Zn2 is also shown extending from the 2-PBImi molecule. The Zn2 metal center also binds to a second set of three dimethylcarbamate molecules. This is illustrated in Figure 2. The second set of dimethylcarbamates, which bridge the Zn2 metal center, are crystallographically unique but structurally similar to those bound to Zn1 (asterisks indicate symmetry equivalent atoms). Figure 3 shows a ball and stick representation of an individual polymer chain to illustrate the chiral behavior of the molecule. The chain propagates along the *c* axis direction. The observation of chirality (the compound crystallizes in the space group *P*3_1_21) is interesting because the structure crystallized from achiral precursors Zn(CH_3_COO_2_)_2_^.^2H_2_O and 2–PBImi. Presumably the sample crystallizes as a equal fraction mixture of *P*3_1_21 and *P*3_2_21 symmetry.

The structure is charge-neutral. The metal to 2-PBImi ligand ratio is 2:1 because each 2-PBImi ligand is shared by the two zinc cations. Therefore, each MBB requires an additional 3- for charge balance. This is accomodated by the three dimethylcarbamate anionic molecules which cap the metals. The structure repeats itself every sixth zinc cluster, resulting in the long 52.470 (11) Å *c* axis.

### Experimental

The reaction mixture containing Zn(CH_3_COO_2_)_2_^.^2H_2_O (0.008 g, 0.0436 mmol) and 2–PBImi (2-phenylbenzimidazole, 0.066 g, 0.3398 mmol) in 3 mL of *N*,*N*'-dimethylformamide (DMF) was placed in a convection oven at 115° C for 72 h inside capped scintillation vials. The capped vials were removed from the oven, and allowed to stand at room temperature over a period of approximately two weeks, after which time pale yellow block-shaped crystals formed.

### Refinement

Hydrogen positions were derived and refined using the riding model within the *SHELXTL* and XSHELL software. Hydrogen atoms were fixed at 0.93 Å and 0.96 Å for aromatic and methyl type C—H bonds, respectively. The Flack (1983) parameter was calculated using 1754 Friedel pairs. The fraction of Friedel pairs measured was approximately 0.67.

### Crystal data

**Table:**

[Zn_2_(C_13_H_9_N_2_)(C_3_H_6_NO_2_)_3_]	*D*_x_ = 1.574 Mg m^−^^3^
*M**_r_* = 588.23	Mo *K*α radiation, λ = 0.71073 Å
Trigonal, *P*3_1_21	Cell parameters from 200 reflections
Hall symbol: P 31 2"	θ = 1–25.0°
*a* = 9.0521 (13) Å	µ = 1.98 mm^−^^1^
*c* = 52.470 (11) Å	*T* = 188 K
*V* = 3723.4 (11) Å^3^	Prism, colourless
*Z* = 6	0.20 × 0.19 × 0.15 mm
*F*(000) = 1812	

### Data collection

**Table:**

Bruker APEX CCD diffractometer	4386 independent reflections
Radiation source: fine-focus sealed tube	4132 reflections with *I* > 2σ(*I*)
graphite	*R*_int_ = 0.044
φ and ω scans	θ_max_ = 25.0°, θ_min_ = 2.3°
Absorption correction: numerical (*SADABS*; Sheldrick, 1996)	*h* = −10→10
*T*_min_ = 0.681, *T*_max_ = 0.742	*k* = −10→10
26966 measured reflections	*l* = −62→62

### Refinement

**Table:**

Refinement on *F*^2^	Secondary atom site location: difference Fourier map
Least-squares matrix: full	Hydrogen site location: inferred from neighbouring sites
*R*[*F*^2^ > 2σ(*F*^2^)] = 0.029	H-atom parameters constrained
*wR*(*F*^2^) = 0.059	*w* = 1/[σ^2^(*F*_o_^2^) + (0.0139*P*)^2^ + 2.9151*P*] where *P* = (*F*_o_^2^ + 2*F*_c_^2^)/3
*S* = 1.09	(Δ/σ)_max_ = 0.001
4386 reflections	Δρ_max_ = 0.25 e Å^−^^3^
333 parameters	Δρ_min_ = −0.24 e Å^−^^3^
0 restraints	Absolute structure: Flack (1983), 1754 Friedel pairs
Primary atom site location: structure-invariant direct methods	Flack parameter: 0.011 (12)

### Special details

**Table:**

Geometry. All e.s.d.'s (except the e.s.d. in the dihedral angle between two l.s. planes) are estimated using the full covariance matrix. The cell e.s.d.'s are taken into account individually in the estimation of e.s.d.'s in distances, angles and torsion angles; correlations between e.s.d.'s in cell parameters are only used when they are defined by crystal symmetry. An approximate (isotropic) treatment of cell e.s.d.'s is used for estimating e.s.d.'s involving l.s. planes.
Refinement. Refinement of *F*^2^ against ALL reflections. The weighted *R*-factor *wR* and goodness of fit *S* are based on *F*^2^, conventional *R*-factors *R* are based on *F*, with *F* set to zero for negative *F*^2^. The threshold expression of *F*^2^ > σ(*F*^2^) is used only for calculating *R*-factors(gt) *etc*. and is not relevant to the choice of reflections for refinement. *R*-factors based on *F*^2^ are statistically about twice as large as those based on *F*, and *R*- factors based on ALL data will be even larger.

### Fractional atomic coordinates and isotropic or equivalent isotropic displacement parameters (Å^2^)

**Table:**

	*x*	*y*	*z*	*U*_iso_*/*U*_eq_	
Zn1	0.61691 (5)	0.56462 (5)	0.031021 (7)	0.02259 (10)	
Zn2	0.88114 (5)	0.62957 (5)	0.139747 (7)	0.02319 (10)	
O1	0.5750 (3)	0.3622 (3)	0.01178 (4)	0.0358 (6)	
O2	0.8274 (3)	0.7528 (3)	0.01830 (4)	0.0354 (6)	
O3	0.5961 (3)	0.4199 (3)	−0.03014 (4)	0.0338 (6)	
O4	0.7517 (3)	0.4560 (3)	0.16515 (4)	0.0436 (7)	
O5	0.9058 (3)	0.5436 (3)	0.20106 (4)	0.0392 (7)	
O6	0.8978 (4)	0.8375 (3)	0.15205 (5)	0.0457 (8)	
N1	0.6497 (3)	0.5170 (3)	0.06693 (4)	0.0211 (6)	
N2	0.7528 (3)	0.5425 (3)	0.10704 (5)	0.0213 (6)	
N3	0.5940 (4)	0.1848 (4)	−0.01588 (5)	0.0345 (8)	
N4	1.0043 (5)	1.0043 (5)	0.0000	0.0365 (11)	
N5	0.6591 (4)	0.2963 (4)	0.20013 (5)	0.0329 (7)	
N6	1.0000	1.1032 (5)	0.1667	0.0411 (12)	
C1	0.7521 (5)	0.8882 (5)	0.07206 (7)	0.0370 (9)	
H1	0.6428	0.8215	0.0655	0.042 (11)*	
C2	0.8316 (6)	1.0654 (5)	0.07046 (8)	0.0474 (11)	
H2	0.7766	1.1172	0.0627	0.042 (10)*	
C3	0.9932 (6)	1.1629 (5)	0.08049 (9)	0.0562 (12)	
H3	1.0462	1.2813	0.0798	0.051 (11)*	
C4	1.0773 (6)	1.0865 (5)	0.09152 (7)	0.0464 (11)	
H4	1.1870	1.1536	0.0979	0.053 (13)*	
C5	0.9985 (5)	0.9104 (5)	0.09305 (6)	0.0338 (9)	
H5	1.0550	0.8595	0.1005	0.029 (10)*	
C6	0.8346 (5)	0.8100 (4)	0.08340 (6)	0.0252 (7)	
C7	0.7473 (4)	0.6227 (4)	0.08569 (6)	0.0220 (7)	
C8	0.6498 (4)	0.3696 (4)	0.10177 (6)	0.0229 (7)	
C9	0.6048 (5)	0.2254 (5)	0.11655 (6)	0.0317 (8)	
H9	0.6466	0.2351	0.1330	0.055 (12)*	
C10	0.4971 (5)	0.0683 (5)	0.10609 (7)	0.0403 (10)	
H10	0.4649	−0.0295	0.1157	0.041 (11)*	
C11	0.4348 (5)	0.0531 (5)	0.08119 (7)	0.0391 (9)	
H11	0.3630	−0.0549	0.0745	0.030 (9)*	
C12	0.4772 (5)	0.1935 (4)	0.06647 (7)	0.0320 (9)	
H12	0.4346	0.1824	0.0500	0.030 (9)*	
C13	0.5861 (4)	0.3535 (4)	0.07689 (6)	0.0240 (7)	
C14	0.8556 (5)	0.8556 (5)	0.0000	0.0238 (11)	
C15	1.1260 (5)	1.0518 (6)	0.02054 (7)	0.0562 (13)	
H15A	1.0726	0.9779	0.0349	0.084*	
H15B	1.1649	1.1677	0.0254	0.084*	
H15C	1.2213	1.0412	0.0150	0.084*	
C16	0.5881 (4)	0.3284 (4)	−0.01132 (6)	0.0274 (8)	
C17	0.5966 (6)	0.1272 (6)	−0.04163 (7)	0.0547 (13)	
H17A	0.5995	0.2086	−0.0536	0.082*	
H17B	0.4961	0.0186	−0.0444	0.082*	
H17C	0.6960	0.1162	−0.0438	0.082*	
C18	0.5903 (7)	0.0760 (6)	0.00490 (8)	0.0587 (13)	
H18A	0.6551	0.1457	0.0190	0.088*	
H18B	0.6388	0.0086	−0.0008	0.088*	
H18C	0.4744	0.0021	0.0101	0.088*	
C19	0.7772 (4)	0.4385 (4)	0.18828 (6)	0.0234 (7)	
C20	0.5019 (5)	0.1739 (6)	0.18801 (8)	0.0565 (13)	
H20A	0.4878	0.2209	0.1724	0.085*	
H20B	0.4078	0.1475	0.1992	0.085*	
H20C	0.5053	0.0717	0.1844	0.085*	
C21	0.6793 (6)	0.2625 (6)	0.22652 (8)	0.0561 (12)	
H21A	0.7903	0.3467	0.2324	0.084*	
H21B	0.6671	0.1512	0.2279	0.084*	
H21C	0.5936	0.2672	0.2367	0.084*	
C22	1.0000	0.9526 (5)	0.1667	0.0290 (12)	
C23	0.8890 (7)	1.1336 (6)	0.15050 (9)	0.0641 (14)	
H23A	0.8378	1.0447	0.1380	0.096*	
H23B	0.9541	1.2417	0.1421	0.096*	
H23C	0.8013	1.1346	0.1607	0.096*	

### Atomic displacement parameters (Å^2^)

**Table:**

	*U*^11^	*U*^22^	*U*^33^	*U*^12^	*U*^13^	*U*^23^
Zn1	0.0257 (2)	0.0255 (2)	0.01562 (18)	0.01208 (19)	−0.00268 (16)	0.00245 (17)
Zn2	0.0258 (2)	0.0255 (2)	0.01727 (18)	0.01204 (18)	−0.00601 (17)	−0.00255 (17)
O1	0.0619 (18)	0.0346 (15)	0.0162 (12)	0.0280 (14)	−0.0006 (12)	−0.0010 (11)
O2	0.0279 (14)	0.0389 (16)	0.0295 (13)	0.0093 (13)	0.0012 (11)	0.0153 (12)
O3	0.0531 (18)	0.0337 (14)	0.0236 (13)	0.0285 (13)	0.0013 (12)	0.0060 (11)
O4	0.0392 (16)	0.0460 (17)	0.0177 (13)	0.0003 (13)	−0.0042 (11)	0.0034 (12)
O5	0.0289 (14)	0.0438 (17)	0.0234 (13)	0.0020 (13)	−0.0076 (11)	0.0032 (12)
O6	0.065 (2)	0.0420 (17)	0.0432 (16)	0.0371 (16)	−0.0322 (15)	−0.0236 (13)
N1	0.0237 (15)	0.0214 (15)	0.0150 (12)	0.0089 (12)	−0.0049 (11)	0.0008 (11)
N2	0.0255 (15)	0.0233 (16)	0.0140 (13)	0.0114 (13)	−0.0020 (11)	0.0008 (11)
N3	0.053 (2)	0.0324 (18)	0.0274 (16)	0.0284 (17)	−0.0003 (14)	0.0015 (14)
N4	0.0324 (18)	0.0324 (18)	0.027 (2)	0.003 (2)	−0.0074 (11)	0.0074 (11)
N5	0.0287 (18)	0.0302 (18)	0.0310 (17)	0.0083 (15)	0.0013 (13)	0.0035 (14)
N6	0.048 (3)	0.0290 (18)	0.053 (3)	0.0238 (15)	−0.019 (2)	−0.0094 (12)
C1	0.038 (2)	0.034 (2)	0.039 (2)	0.0182 (19)	−0.0060 (19)	0.0007 (18)
C2	0.056 (3)	0.033 (2)	0.056 (3)	0.024 (2)	−0.008 (2)	0.005 (2)
C3	0.069 (3)	0.018 (2)	0.073 (3)	0.015 (2)	−0.009 (3)	0.001 (2)
C4	0.047 (3)	0.032 (2)	0.042 (2)	0.006 (2)	−0.011 (2)	−0.003 (2)
C5	0.036 (2)	0.031 (2)	0.0310 (19)	0.0140 (19)	−0.0028 (17)	0.0045 (17)
C6	0.032 (2)	0.0218 (18)	0.0216 (16)	0.0135 (16)	−0.0013 (15)	−0.0028 (15)
C7	0.0218 (17)	0.0267 (19)	0.0179 (15)	0.0124 (16)	−0.0007 (13)	−0.0016 (15)
C8	0.0258 (18)	0.0242 (19)	0.0212 (17)	0.0143 (16)	−0.0003 (14)	0.0013 (14)
C9	0.043 (2)	0.033 (2)	0.0199 (17)	0.0193 (19)	−0.0058 (16)	0.0012 (16)
C10	0.058 (3)	0.026 (2)	0.031 (2)	0.017 (2)	0.0017 (18)	0.0065 (17)
C11	0.042 (2)	0.027 (2)	0.037 (2)	0.0088 (19)	−0.005 (2)	−0.0027 (18)
C12	0.035 (2)	0.025 (2)	0.0264 (19)	0.0077 (17)	−0.0073 (16)	−0.0012 (15)
C13	0.0241 (18)	0.0249 (19)	0.0170 (16)	0.0077 (16)	−0.0013 (14)	0.0007 (14)
C14	0.026 (2)	0.026 (2)	0.023 (2)	0.015 (2)	0.0012 (10)	−0.0012 (10)
C15	0.034 (3)	0.065 (3)	0.039 (2)	0.001 (2)	−0.012 (2)	0.006 (2)
C16	0.025 (2)	0.026 (2)	0.0276 (19)	0.0097 (17)	0.0011 (15)	0.0037 (15)
C17	0.089 (4)	0.061 (3)	0.031 (2)	0.051 (3)	−0.005 (2)	−0.011 (2)
C18	0.101 (4)	0.050 (3)	0.046 (3)	0.054 (3)	0.015 (3)	0.013 (2)
C19	0.0269 (19)	0.0233 (18)	0.0231 (18)	0.0150 (17)	0.0023 (15)	0.0000 (15)
C20	0.041 (3)	0.044 (3)	0.049 (3)	−0.006 (2)	0.006 (2)	0.000 (2)
C21	0.054 (3)	0.066 (3)	0.047 (3)	0.029 (3)	0.008 (2)	0.028 (2)
C22	0.025 (3)	0.022 (2)	0.040 (3)	0.0127 (14)	−0.002 (2)	−0.0010 (12)
C23	0.098 (4)	0.066 (3)	0.060 (3)	0.065 (3)	−0.026 (3)	−0.016 (3)

### Geometric parameters (Å, °)

**Table:**

Zn1—O2	1.932 (2)	C3—C4	1.386 (6)
Zn1—O3^i^	1.942 (2)	C3—H3	0.9300
Zn1—O1	1.956 (2)	C4—C5	1.385 (5)
Zn1—N1	1.988 (2)	C4—H4	0.9300
Zn2—O6	1.923 (3)	C5—C6	1.391 (5)
Zn2—O5^ii^	1.933 (2)	C5—H5	0.9300
Zn2—O4	1.944 (2)	C6—C7	1.474 (4)
Zn2—N2	2.000 (3)	C8—C9	1.393 (5)
O1—C16	1.270 (4)	C8—C13	1.405 (4)
O2—C14	1.271 (3)	C9—C10	1.374 (5)
O3—C16	1.267 (4)	C9—H9	0.9300
O3—Zn1^i^	1.942 (2)	C10—C11	1.402 (5)
O4—C19	1.260 (4)	C10—H10	0.9300
O5—C19	1.266 (4)	C11—C12	1.368 (5)
O5—Zn2^ii^	1.933 (2)	C11—H11	0.9300
O6—C22	1.251 (3)	C12—C13	1.393 (5)
N1—C7	1.349 (4)	C12—H12	0.9300
N1—C13	1.394 (4)	C14—O2^i^	1.271 (3)
N2—C7	1.349 (4)	C15—H15A	0.9600
N2—C8	1.391 (4)	C15—H15B	0.9600
N3—C16	1.349 (4)	C15—H15C	0.9600
N3—C17	1.452 (4)	C17—H17A	0.9600
N3—C18	1.458 (5)	C17—H17B	0.9600
N4—C14	1.346 (6)	C17—H17C	0.9600
N4—C15^i^	1.444 (4)	C18—H18A	0.9600
N4—C15	1.445 (4)	C18—H18B	0.9600
N5—C19	1.346 (4)	C18—H18C	0.9600
N5—C20	1.443 (5)	C20—H20A	0.9600
N5—C21	1.450 (5)	C20—H20B	0.9600
N6—C22	1.363 (6)	C20—H20C	0.9600
N6—C23^ii^	1.442 (5)	C21—H21A	0.9600
N6—C23	1.442 (5)	C21—H21B	0.9600
C1—C6	1.393 (5)	C21—H21C	0.9600
C1—C2	1.394 (5)	C22—O6^ii^	1.251 (3)
C1—H1	0.9300	C23—H23A	0.9600
C2—C3	1.380 (6)	C23—H23B	0.9600
C2—H2	0.9300	C23—H23C	0.9600
			
O2—Zn1—O3^i^	115.83 (11)	C8—C9—H9	121.0
O2—Zn1—O1	106.95 (11)	C9—C10—C11	121.1 (3)
O3^i^—Zn1—O1	111.27 (11)	C9—C10—H10	119.4
O2—Zn1—N1	109.29 (10)	C11—C10—H10	119.4
O3^i^—Zn1—N1	107.59 (10)	C12—C11—C10	121.5 (4)
O1—Zn1—N1	105.43 (10)	C12—C11—H11	119.2
O6—Zn2—O5^ii^	116.22 (13)	C10—C11—H11	119.2
O6—Zn2—O4	106.34 (12)	C11—C12—C13	117.9 (3)
O5^ii^—Zn2—O4	110.93 (12)	C11—C12—H12	121.0
O6—Zn2—N2	114.79 (11)	C13—C12—H12	121.0
O5^ii^—Zn2—N2	102.32 (10)	C12—C13—N1	131.2 (3)
O4—Zn2—N2	105.86 (11)	C12—C13—C8	120.9 (3)
C16—O1—Zn1	136.1 (2)	N1—C13—C8	107.9 (3)
C14—O2—Zn1	130.9 (2)	O2—C14—O2^i^	124.4 (4)
C16—O3—Zn1^i^	129.2 (2)	O2—C14—N4	117.8 (2)
C19—O4—Zn2	133.4 (2)	O2^i^—C14—N4	117.8 (2)
C19—O5—Zn2^ii^	135.6 (2)	N4—C15—H15A	109.5
C22—O6—Zn2	133.2 (3)	N4—C15—H15B	109.5
C7—N1—C13	104.8 (2)	H15A—C15—H15B	109.5
C7—N1—Zn1	130.8 (2)	N4—C15—H15C	109.5
C13—N1—Zn1	123.9 (2)	H15A—C15—H15C	109.5
C7—N2—C8	104.8 (3)	H15B—C15—H15C	109.5
C7—N2—Zn2	132.1 (2)	O3—C16—O1	124.6 (3)
C8—N2—Zn2	123.0 (2)	O3—C16—N3	118.3 (3)
C16—N3—C17	121.8 (3)	O1—C16—N3	117.1 (3)
C16—N3—C18	121.3 (3)	N3—C17—H17A	109.5
C17—N3—C18	116.9 (3)	N3—C17—H17B	109.5
C14—N4—C15^i^	122.0 (2)	H17A—C17—H17B	109.5
C14—N4—C15	122.0 (2)	N3—C17—H17C	109.5
C15^i^—N4—C15	116.0 (5)	H17A—C17—H17C	109.5
C19—N5—C20	122.6 (3)	H17B—C17—H17C	109.5
C19—N5—C21	121.3 (3)	N3—C18—H18A	109.5
C20—N5—C21	116.0 (3)	N3—C18—H18B	109.5
C22—N6—C23^ii^	122.6 (2)	H18A—C18—H18B	109.5
C22—N6—C23	122.6 (2)	N3—C18—H18C	109.5
C23^ii^—N6—C23	114.8 (5)	H18A—C18—H18C	109.5
C6—C1—C2	120.7 (4)	H18B—C18—H18C	109.5
C6—C1—H1	119.6	O4—C19—O5	125.0 (3)
C2—C1—H1	119.6	O4—C19—N5	117.2 (3)
C3—C2—C1	119.0 (4)	O5—C19—N5	117.8 (3)
C3—C2—H2	120.5	N5—C20—H20A	109.5
C1—C2—H2	120.5	N5—C20—H20B	109.5
C2—C3—C4	120.7 (4)	H20A—C20—H20B	109.5
C2—C3—H3	119.6	N5—C20—H20C	109.5
C4—C3—H3	119.6	H20A—C20—H20C	109.5
C5—C4—C3	120.2 (4)	H20B—C20—H20C	109.5
C5—C4—H4	119.9	N5—C21—H21A	109.5
C3—C4—H4	119.9	N5—C21—H21B	109.5
C4—C5—C6	119.9 (4)	H21A—C21—H21B	109.5
C4—C5—H5	120.0	N5—C21—H21C	109.5
C6—C5—H5	120.0	H21A—C21—H21C	109.5
C5—C6—C1	119.4 (3)	H21B—C21—H21C	109.5
C5—C6—C7	120.3 (3)	O6—C22—O6^ii^	124.9 (5)
C1—C6—C7	120.3 (3)	O6—C22—N6	117.6 (2)
N1—C7—N2	114.3 (3)	O6^ii^—C22—N6	117.6 (2)
N1—C7—C6	122.7 (3)	N6—C23—H23A	109.5
N2—C7—C6	122.9 (3)	N6—C23—H23B	109.5
N2—C8—C9	131.4 (3)	H23A—C23—H23B	109.5
N2—C8—C13	108.1 (3)	N6—C23—H23C	109.5
C9—C8—C13	120.5 (3)	H23A—C23—H23C	109.5
C10—C9—C8	118.1 (3)	H23B—C23—H23C	109.5
C10—C9—H9	121.0		
			
O2—Zn1—O1—C16	−41.4 (4)	C7—N2—C8—C13	0.2 (4)
O3^i^—Zn1—O1—C16	86.0 (4)	Zn2—N2—C8—C13	−177.1 (2)
N1—Zn1—O1—C16	−157.7 (3)	N2—C8—C9—C10	179.2 (3)
O3^i^—Zn1—O2—C14	−36.3 (3)	C13—C8—C9—C10	−0.1 (5)
O1—Zn1—O2—C14	88.3 (3)	C8—C9—C10—C11	0.4 (6)
N1—Zn1—O2—C14	−158.0 (2)	C9—C10—C11—C12	−0.7 (6)
O6—Zn2—O4—C19	−62.3 (4)	C10—C11—C12—C13	0.5 (6)
O5^ii^—Zn2—O4—C19	64.9 (4)	C11—C12—C13—N1	−179.2 (4)
N2—Zn2—O4—C19	175.1 (3)	C11—C12—C13—C8	−0.2 (5)
O5^ii^—Zn2—O6—C22	−37.6 (3)	C7—N1—C13—C12	179.3 (4)
O4—Zn2—O6—C22	86.4 (3)	Zn1—N1—C13—C12	−7.3 (6)
N2—Zn2—O6—C22	−156.9 (3)	C7—N1—C13—C8	0.2 (4)
O2—Zn1—N1—C7	33.4 (3)	Zn1—N1—C13—C8	173.6 (2)
O3^i^—Zn1—N1—C7	−93.2 (3)	N2—C8—C13—C12	−179.5 (3)
O1—Zn1—N1—C7	148.0 (3)	C9—C8—C13—C12	−0.1 (5)
O2—Zn1—N1—C13	−138.1 (3)	N2—C8—C13—N1	−0.3 (4)
O3^i^—Zn1—N1—C13	95.4 (3)	C9—C8—C13—N1	179.2 (3)
O1—Zn1—N1—C13	−23.5 (3)	Zn1—O2—C14—O2^i^	−19.90 (16)
O6—Zn2—N2—C7	39.0 (3)	Zn1—O2—C14—N4	160.10 (16)
O5^ii^—Zn2—N2—C7	−87.8 (3)	C15^i^—N4—C14—O2	177.0 (3)
O4—Zn2—N2—C7	156.0 (3)	C15—N4—C14—O2	−3.0 (3)
O6—Zn2—N2—C8	−144.5 (2)	C15^i^—N4—C14—O2^i^	−3.0 (3)
O5^ii^—Zn2—N2—C8	88.7 (3)	C15—N4—C14—O2^i^	177.0 (3)
O4—Zn2—N2—C8	−27.5 (3)	Zn1^i^—O3—C16—O1	−8.1 (5)
C6—C1—C2—C3	−0.6 (6)	Zn1^i^—O3—C16—N3	172.0 (2)
C1—C2—C3—C4	1.4 (7)	Zn1—O1—C16—O3	−18.9 (6)
C2—C3—C4—C5	−1.2 (7)	Zn1—O1—C16—N3	161.0 (3)
C3—C4—C5—C6	0.2 (6)	C17—N3—C16—O3	−4.6 (6)
C4—C5—C6—C1	0.6 (5)	C18—N3—C16—O3	178.3 (4)
C4—C5—C6—C7	−178.0 (3)	C17—N3—C16—O1	175.5 (4)
C2—C1—C6—C5	−0.4 (6)	C18—N3—C16—O1	−1.7 (6)
C2—C1—C6—C7	178.2 (3)	Zn2—O4—C19—O5	1.1 (6)
C13—N1—C7—N2	−0.1 (4)	Zn2—O4—C19—N5	−178.3 (3)
Zn1—N1—C7—N2	−172.8 (2)	Zn2^ii^—O5—C19—O4	−7.3 (6)
C13—N1—C7—C6	−177.6 (3)	Zn2^ii^—O5—C19—N5	172.1 (3)
Zn1—N1—C7—C6	9.7 (5)	C20—N5—C19—O4	−5.1 (5)
C8—N2—C7—N1	0.0 (4)	C21—N5—C19—O4	178.3 (4)
Zn2—N2—C7—N1	176.9 (2)	C20—N5—C19—O5	175.5 (4)
C8—N2—C7—C6	177.5 (3)	C21—N5—C19—O5	−1.1 (5)
Zn2—N2—C7—C6	−5.6 (5)	Zn2—O6—C22—O6^ii^	−15.56 (19)
C5—C6—C7—N1	−140.8 (3)	Zn2—O6—C22—N6	164.44 (19)
C1—C6—C7—N1	40.7 (5)	C23^ii^—N6—C22—O6	179.5 (3)
C5—C6—C7—N2	41.9 (5)	C23—N6—C22—O6	−0.5 (3)
C1—C6—C7—N2	−136.6 (3)	C23^ii^—N6—C22—O6^ii^	−0.5 (3)
C7—N2—C8—C9	−179.1 (4)	C23—N6—C22—O6^ii^	179.5 (3)
Zn2—N2—C8—C9	3.6 (5)		

Symmetry codes: (i) *y*, *x*, −*z*; (ii) −*x*+2, −*x*+*y*+1, −*z*+1/3.
